# Survey of healthcare experiences of Australian adults living with rare diseases

**DOI:** 10.1186/s13023-016-0409-z

**Published:** 2016-03-24

**Authors:** Caron Molster, Debra Urwin, Louisa Di Pietro, Megan Fookes, Dianne Petrie, Sharon van der Laan, Hugh Dawkins

**Affiliations:** Office of Population Health Genomics, Department of Health, Perth, WA Australia; Genetic Support Network Victoria, Melbourne, VIC Australia; Rare Voices Australia, Sydney, NSW Australia; Genetic Alliance Australia, Sydney, NSW Australia; Genetic and Rare Diseases Network, Perth, WA Australia; Centre for Population Health Research, Curtin University of Technology, Bentley, WA Australia; School of Pathology and Laboratory Medicine, University of Western Australia, Nedlands, WA Australia; Centre for Comparative Genomics, Murdoch University, Murdoch, WA Australia

**Keywords:** Healthcare, Survey, Australia, Experiences, Diagnosis, Health services, Rare diseases

## Abstract

**Background:**

Few studies have examined whether the healthcare needs of people living with rare diseases are being met. This study explores the experiences of Australian adults living with rare diseases in relation to diagnosis, information provision at the time of diagnosis, use of health and support services and involvement in research on their condition.

**Methods:**

The survey respondents are self-selected from the population of Australian residents aged 18 years and over who are living with a rare disease. An online survey was implemented between July-August 2014. Purposive snowballing sampling was used. The results are reported as percentages with significant differences between sub-groups assessed using chi-squared analyses.

**Results:**

Eight hundred ten responses were obtained from adults living with a rare disease. 92.1 % had a confirmed diagnosis, of which 30.0 % waited five or more years for a diagnosis, 66.2 % had seen three or more doctors to get a diagnosis and 45.9 % had received at least one incorrect diagnosis. Almost three quarters (72.1 %) received no or not enough information at the time of diagnosis. In the 12 months prior to the survey, over 80 % of respondents had used the services of a general practitioner and a medical specialist while around a third had been inpatients at a hospital or had visited an emergency department. Only 15.4 % of respondents had ever used paediatric services, 52.8 % of these had experienced problems in the transition from paediatric to adult services. Only 20.3 % knew of a patient registry for their condition and 24.8 % were informed of clinical trials.

**Conclusions:**

These findings suggest that not all healthcare needs of people living with rare diseases are being met. Structural changes to Australian healthcare systems may be required to improve the integration and coordination of diagnosis and care. Health professionals may need greater awareness of rare diseases to improve the diagnostic process and support to meet the information requirements of people newly diagnosed with rare diseases. Health service use is likely higher than for the general population and further epidemiological studies are needed on the impact of rare diseases on the healthcare system.

## Background

In terms of prevalence, a rare disease has been defined as one that is present in fewer than one person in 2000 in a population [1, 2]. The range of rare diseases is very broad and around 80 % are thought to be genetic in origin [1, 3]. For example *Alopecia areata* is a complex genetic, immune mediated disease that results in partial to universal hair loss [4], while *Kabuki syndrome* is caused by a single mutation in one of two genes, and results in multiple congenital anomalies such as altered facial features, skeletal anomalies, growth deficiency and intellectual disability [5]. Although the aetiology and symptoms show great diversity, there are some commonalities across the range of rare diseases. For example, rare diseases often involve multi system dysfunction, require complex care, have no effective treatment, and are incurable [6–8]. Many are also associated with motor, sensory or intellectual impairment [9] and impose significant social, emotional and financial burdens on people living with rare diseases, and their carers and families [10, 11].

Stakeholder consultations with members of the Australian rare diseases community have indicated that common features across the range of rare diseases lead to similar needs from the health system [12, 13]. This includes access to a wide range of health and social support services across primary, secondary and tertiary sectors [13, 14], along with:

- timely and accurate diagnosis;
- timely post-diagnosis information to inform decision-making about ongoing care;
- complex case management which requires access to a range of specialists, services and programs;
- coordinated and integrated care so that there are no gaps in service delivery across the lifespan, including transitions from paediatric to adult care;
- access to health professionals who are aware of, experienced with and knowledgeable about rare diseases; and
- access to infrastructure to support clinical, epidemiological and translational research, such as patient registries.

There are few studies that have examined whether such healthcare needs are being met for people living with rare diseases. Two studies conducted in the United Kingdom (UK) [10, 15] and Europe [8, 16] identify experiences of delayed diagnosis, misdiagnosis, a lack of information provided at the time of diagnosis, a lack of care coordination, problems with transition from paediatric to adult care and the prescription of incorrect medications. Anecdotally, Australians living with rare diseases have described similar experiences to those identified internationally. Yet to date there has only been a single published study with a small sample size and coverage restricted to paediatric patients diagnosed with genetic metabolic disorders. This study surveyed 30 families as a pilot study and found that 52 % had consulted three or more doctors before receiving a correct diagnosis and 43 % felt diagnosis was delayed [14].

The purpose of the present study was to explore the healthcare experiences of adults living with a rare disease in Australia. The Australian healthcare system includes a mix of public and private health services. There are no officially recognised centres of expertise for rare diseases, although some disease-specific clinics do exist. There is no national plan for rare diseases and only one state, Western Australia, has adopted a jurisdictional strategic framework for rare diseases [17]. Within this context, the objectives of the study were to examine experiences of diagnosis, perceptions of the information provided at the time of diagnosis, availability and use of health and support services, experiences during the transition from paediatric to adult health services and involvement in research.

## Methods

### Survey instrument

**Fig. 1:**
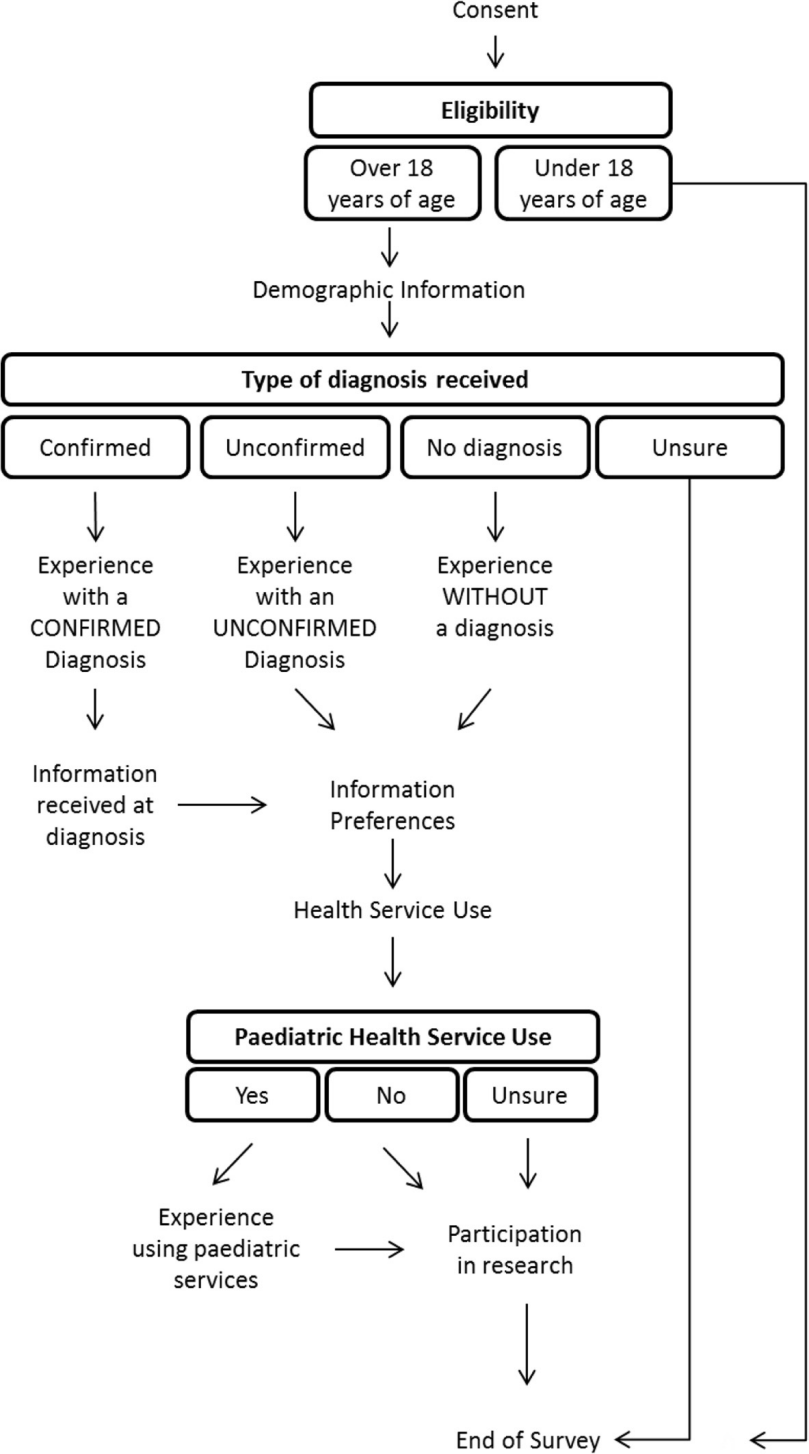
Schematic diagram of the survey instrument. Compulsory questions that lead participants to be skipped to particular sections within the survey, together with their possible answers are shown in boxes

The survey was hosted online using the SurveyMonkey platform (SurveyMonkey Inc. Palo Alto, USA). The survey instrument was adapted with permission from the survey developed by Rare Diseases UK [10, 15]. Some modifications were made to ensure that the questions were relevant in the Australian context, particularly relating to the primary, secondary and tertiary structure of the Australian healthcare system. A schematic diagram of the survey instrument is provided at Fig. 1. The full survey instrument is available from the authors upon request.

The survey instrument contains a majority of quantitative, close-ended questions with defined response categories. A smaller number of questions asked participants to provide qualitative information and for these an open-ended text box was provided. At the conclusion of each survey section, participants were also given the opportunity to provide any other comments they had on the section topic. Only data from the quantitative questions are reported in this paper.

The first section of the survey was used to obtain consent and to determine participant eligibility. Participants were provided with information about the survey, including the purpose, the agencies conducting the study and assurance around anonymity and aggregation of data for reporting purposes. They were then advised that by clicking the “next” button they were consenting to participate in the survey. As the survey was completed anonymously, once data was submitted by clicking the “submit” button at the end of the survey it was not possible to withdraw individual respondents’ data.

The demographic section of the survey asked participants for information including gender, age and state of residence, the rare disease being lived with and the type of diagnosis that had been received (i.e., confirmed, unconfirmed or no diagnosis). Participants were also asked to rate their health, on a 5 point scale from excellent to poor. This question has previously been shown to be a reliable measure of general health [18] and is significantly associated with the relative risk of mortality [19].

The main body of the survey had four sections measuring key aspects of the health care experience, namely experiences of diagnosis, access to and use of health and support services, transition from paediatric to adult care settings and participation in research.

### Survey sampling

Those eligible to complete the survey were Australian residents aged 18 years or older who are living with a rare disease. Carers or paid support workers of people meeting these criteria were also invited to complete the survey, if the person living with a rare disease did not have the intellectual and/or physical capability to complete the survey themselves. The study was limited to adults living with rare diseases for two reasons. Firstly, the authors were aware that a survey of the healthcare experiences of children was being conducted simultaneously and did not want to over-burden the families and carers of children living with rare diseases with two similar surveys. Secondly, targeting the adult population enabled us to examine issues outside the paediatric healthcare system such as diagnosis and transition to using adult health services.

To our knowledge a complete sample frame of all adults living with a rare disease in Australia does not exist. Unknown is the total number of people living with a rare disease, their contact details and their socio-demographic characteristics such as age, gender, highest education level and location of residence. For these reasons, purposive snowball sampling was used for this study. A link to the online survey was distributed via the networks and mailing lists of the Office of Population Health Genomics, within the Western Australian Department of Health and the four peak bodies in the Australian rare and genetic disease sector, namely Rare Voices Australia, Genetic Alliance Australia, Genetic Support Network Victoria and the Genetic and Rare Diseases Network. The survey link was initially distributed to over 300 patient support groups across Australia, as well as individuals registered with any of the collaborating organisations. Recipients of emails were asked to forward the link on to other people who were eligible for the survey. Because of the potential for participants to receive more than one invitation to participate, survey responses were limited to one per computer. Data were collected over a six week period during July-August 2014.

### Data analysis

SAS 9.2 survey analysis procedures for frequency tables were used to analyse the survey data (SAS Institute Inc. Cary, USA). The results are reported as percentages. Differences in responses associated with demographics were assessed for significance using chi-squared analyses.

## Results

### Sample characteristics

In total 1014 completed surveys were received. These were initially examined to confirm whether or not the diseases that respondents had reported having were rare. Alternative names and spellings were checked by referring to the Orphanet database of rare diseases (www.orpha.net). If the disease was listed in Orphanet with a prevalence of 1 in 2000 or less the respondent was deemed to be living with a rare disease. On this basis, 810 respondents were considered to be living with a rare disease. Most of the remaining respondents (*n* = 172) were living with a disease that typically has similar characteristics to a rare disease in that it is chronic and debilitating, but is more prevalent than 1 in 2000 people in the population. Others were removed from the study due to not meeting the age criteria, that is, they were aged less than 18 years.

Of the 810 respondents who were considered to be living with a rare disease, 92 % had received a confirmed diagnosis. For the purposes of this study, a confirmed diagnosis is defined as a diagnosis that the doctor and patient are certain is correct based on a genetic (or other) test or clinical manifestation. Six percent had an unconfirmed diagnosis, defined as a diagnosis that the doctor and patient think is most likely to cause the symptoms experienced, but are not certain. The remainder had no diagnosis (1.5 %) or were unsure (0.5 %). In order to ensure we were examining the healthcare experiences of adults living with a rare disease, only those respondents with a confirmed diagnosis of a rare disease are included in the findings reported below.

Table 1 shows the demographic characteristics of survey respondents living with a confirmed rare disease. Responses were received from people located in all states and territories of Australia, and their numbers were generally in proportion to the size of the population in each state and territory [20]. The age of respondents ranged from 18 to 87 years of age, with a median age of 47 years. The majority of respondents were female (76.3 %) and were themselves living with a rare disease (89.2 %), as opposed to being a relative/carer (10.0 %) or paid support worker (0.8 %). Almost one in five (18.6 %) reported their health as poor. Respondents reported living with up to four different rare diseases although 84 % of respondents reported living with only one rare disease (Table 1).

**Table 1:** Characteristics of the survey response group

	Number of people	% of Response
Sex (*n* = 744)		
Female	568	76.3
Male	176	23.7
Age groups (*n* = 234)		
18–34 years	61	26.1
35–64 years	129	55.1
65 years and over	44	18.8
State of residence (*n* = 741)		
New South Wales	212	28.6
Victoria	182	24.6
Queensland	159	21.5
Western Australia	92	12.4
South Australia	44	5.9
Australian Capital Territory	25	3.4
Tasmania	23	3.1
Northern Territory	4	0.5
Role of person answering the survey (*n* = 741)		
A paid support worker	6	0.8
A person living with a rare disease.	661	89.2
A relative/carer of a person with a rare disease	74	10.0
Other	0	0
Number of rare diseases per person (*n* = 746)		
1	628	84.2
2	93	12.5
3	21	2.8
4	4	0.5
Self-reported health status (*n* = 743)		
Excellent	25	3.4
Very good	113	15.2
Good	211	28.4
Fair	256	34.5
Poor	138	18.6

A total of 185 rare diseases were represented (sub-types of a rare disease were combined together). Ninety-nine diseases had only one respondent, while the greatest number of responses for a single disease group (*n* = 52) was for Ehlers-Danlos syndrome, which has multiple sub-types. Table 2 provides a list of the diseases represented in the survey, as organised by nosological group based on the linearized Orphanet classifications [21]. The survey outcomes were not analysed by nosological disease groups, since there were 21 groups of diseases and the size of the sample in some groups was too small to detect significant differences in responses.

**Table 2:** Diseases represented in the confirmed rare disease group

Rare disease groups (*n* = 746)	Number of people	% of Response	Diseases in group
Rare abdominal surgical disease	1	0.1	1
Rare allergic diseases	17	2.3	1
Rare bone disease	5	0.7	4
Rare circulatory disease	1	0.1	1
Rare developmental defect during embryogenesis	80	10.7	37
Rare endocrine diseases	39	5.2	10
Rare eye disease	1	0.1	1
Rare gastroenterologic disease	21	2.8	6
Rare gynecologic or obstetric disease	1	0.1	1
Rare hematologic disease	21	2.8	7
Rare hepatic disease	9	1.2	2
Rare immune disease	41	5.5	7
Rare inborn errors of metabolism	50	6.7	13
Rare neoplastic disease	11	1.5	8
Rare neurological disease	263	35.3	45
Rare otorhinolaryngologic disease	3	0.4	1
Rare renal disease	6	0.8	2
Rare respiratory disease	55	7.4	4
Rare skin disease	22	3.0	12
Rare systemic or rheumatologic disease	90	12.1	19
Unclassified	9	1.2	3

### Experiences of diagnosis

Table 3 shows that of the respondents with a confirmed diagnosis, 17.4 % had been diagnosed as children, that is between birth and 17 years of age, although this was higher for men compared to women (24.6 % versus 15.0 %, *p* < 0.01). A quarter (25.2 %) of respondents were diagnosed within three months of first seeking medical help, although this was higher for those diagnosed as children compared to those diagnosed as adults (43.4 % versus 21.8 %, *p* < 0.001). Around half the respondents (51.2 %) waited 1 year or more for a diagnosis, with almost one-third (30.0 %) waiting five or more years.

**Table 3:** Experiences of diagnosis

	Number of people	% of Response
Age at diagnosis (*n* = 732)		
0–17 years	127	17.4
18 years or more	605	82.6
Time to diagnosis (*n* = 718)		
<3 months	181	25.2
3–12 months	169	23.5
1–5 years	153	21.2
5–10 years	147	20.5
>20 years	68	9.5
Number of doctors seen to get a confirmed diagnosis (*n* = 735)		
1–2	248	33.7
3–5	275	37.4
6–10	122	16.6
11 or more	90	12.2
Incorrect diagnosis (*n* = 737)		
At least one incorrect diagnosis	338	45.9
No incorrect diagnosis	360	48.8
Unsure	39	5.3
Information on condition received (*n* = 742)		
Received no information	141	19.0
Received some information but not enough	394	53.1
Received enough information	192	25.9
Unsure	15	2.0

Two-thirds of respondents (66.2 %) had consulted three or more doctors to get a confirmed diagnosis (Table 3). The percentage was lower for people diagnosed as children compared to those diagnosed as adults (50.4 % versus 78.6 %, *p* < 0.001) and for men compared to women (57.9 % versus 68.2 %, *p* < 0.05). Almost half of the respondents (45.9 %) had received at least one incorrect diagnosis, that is, they were initially told they had a condition but were subsequently told they did not have that condition. This occurrence of incorrect diagnosis was lower for respondents diagnosed as children compared to those diagnosed as adults (35.6 % versus 51.2 %, *p* < 0.01).

### Information provision at the time of diagnosis

Around one in five respondents (19.0 %) had not received any information about their condition at the time of diagnosis (Table 3). Of the respondents who received information, 50.0 % indicated that they understood all of the information provided (Table 4). The vast majority of those who received information were given this information by a medical specialist (82.9 %). Respondents who received information from a general practitioner (GP) were less likely to say they received enough information (16.4 %, *p* < 0.01) while those who received information from a genetic counsellor were more likely to say they received enough information (43.9 %, *p* < 0.05).

**Table 4:** Information received at the time of diagnosis^a^

	Number of people	% of Response
Understanding of information provided (*n* = 578)		
Did not understand all the information	289	50.0
Understood all the information	289	50.0
Who provided information^b^ (*n* = 578)		
General practitioner	73	12.5
Medical specialist	486	82.9
Genetic counsellor	82	14
Allied health professional	16	2.7
Other	42	7.2
Format of information provided at diagnosis^b^ (*n* = 578)		
Printed material (e.g., brochures, leaflet)	188	32.1
Referred to website	156	26.6
Social media	87	14.8
Journal article	79	13.5
Verbal only^c^	183	31.2
Other	9	1.5

^a^Among respondents who received information at the time of diagnosis (*n* = 586)

^b^Multiple responses reported

^c^Respondents were not given this option to choose. Instead it was nominated as a response to the option ‘other’

Almost one-third of respondents reported receiving verbal information only (31.2 %) or printed material (32.1 %) and 26.6 % received a referral to a website. Those who received verbal information only were less likely to report they had understood all of the information provided (42.8 %, *p* < 0.05) and that they received enough information (21.9 %, *p* < 0.001), while people referred to a website were more likely to report they had understood all the information they were given (64.7 %, *p* < 0.001) and that they had received enough information (47.4 %, *p* < 0.001). Compared to respondents who received only one source of information, those provided with two or more sources of information were more likely to report the information provided was enough (43.3 % versus 22.1 %, *p* < 0.001) and that it was understood (61.2 % versus 46.7 %, *p* < 0.01).

### Preferred information formats, information sources, knowledge and the use of support groups

Of the options provided in the survey, 77.6 % percent of respondents reported that referral to a website was the most preferred format of information on their condition (Table 5), followed by social media (56.2 %), printed materials such as brochures or leaflets (52.4 %) and journal articles (49.3 %).

**Table 5:** Information, knowledge and use of support groups

	Number of people	% of Response
Preferred format for information^a^ (*n* = 746)		
Referred to website	579	77.6
Social media	419	56.2
Printed material (e.g., brochures, leaflet)	391	52.4
Journal article	368	49.3
Book	257	34.5
DVD	188	25.2
Other	67	9.0
Main source of information^a^ (*n* = 746)		
Medical specialist	453	60.7
Patient organisation	291	39.0
Other people or families	262	35.1
Own research	112	15.0
GP	86	11.5
Genetic councilor	44	5.9
Allied health professional	38	5.1
Other	202	27.1
Now have sufficient knowledge of condition (*n* = 744)		
Yes	437	58.7
No	203	27.3
Don’t know	104	14.0
Have a specific person to ask questions about condition (*n* = 737)		
Yes	438	59.4
No	299	40.6
Have used a patient support group in past 12 months (*n* = 620)		
Daily	62	10.0
Weekly	47	7.6
Monthly	53	8.6
Several times	98	15.8
Once or twice	72	11.6
Never	288	46.5

^a^Multiple responses reported

At the time of the survey, the main sources of information available to respondents on their condition were medical specialists (60.7 %), patient organisations (39.0 %) and other people or families (35.1 %, Table 5). Six in ten respondents (59.4 %) had a specific person they could consult with questions on their condition however 27.3 % did not think they now have sufficient knowledge of their condition. Of those who did have someone to ask questions, the vast majority said this person is a medical specialist. In the 12 months prior to the survey, 53.5 % of respondents had used the services of a patient organisation or support group.

### Access to and use of healthcare and other services

Table 6 shows that the majority of respondents agreed that they received sufficient medical support (66.4 %) but fewer agreed they received sufficient social (34.1 %), financial (14.5 %) and psychological (20.6 %) support. In the 12 months prior to the survey, 8 in 10 respondents had used the services of a GP (81.1 %) and a medical specialist (81.4 %) at least once (Table 7), 40.3 % had used an outpatients service or clinic, while around a third had been inpatients at a hospital (28.0 %) or had visited an emergency department (33.4 %). This level of service use is higher than that reported for the general population of Australians aged 15 years and older [22], where 35 % had visited a medical specialist, 9 % had used an outpatient service, 13 % had been an inpatient at hospital and 12 % had used an emergency department.

**Table 6:** Received sufficient support

	Agree	Neither agree nor disagree	Disagree	Don’t know
Medical (*n* = 624)	66.4	18.9	14.6	–
Social (*n* = 709)	34.1	25.4	40.6	–
Financial (*n* = 725)	14.5	21.1	61.0	3.4
Psychological (*n* = 727)	20.6	26.3	50.3	2.8

**Table 7:** Health services used at least once in the 12 months prior to survey

Service used	n^a^	% of total sample	Median^b^	Mode^b^	Range^b^
General Practitioner	605	81.1	6	2	1–100
Allied Health	286	38.3	5	2	1–250
Medical Specialist	607	81.4	2	2	1–365
Emergency Department	249	33.4	4	1	1–32
Hospital Outpatients/Clinics	301	40.3	4	1	1–100
Hospital Inpatients	209	28.0	2	1	1–365
Dental Services	236	31.6	2	1	1–31
Mental Health Services	207	27.7	5	1	1–52
Alternative Health Services	173	23.2	5	1	1–175

^a^The number of respondents living with a confirmed diagnosis who used a service at least once

^b^Among those who used the service at least once

Nearly four in ten (38.8 %) consulted three or more specialists for their ongoing care yet only 9.2 % had a designated care coordinator (Table 8). Similarly, 39.1 % travelled more than 50 km to see their medical specialists, while only 3.9 % had used telehealth services. One-fifth (22.1 %) knew of a specialist centre for their condition and the majority of these respondents had used that centre. Overall, 37.0 % were satisfied with the adult services they used while 25.2 % were dissatisfied and 37.8 % neither satisfied nor dissatisfied.

**Table 8:** Health service experiences

	Number of people	% of Response
Number of specialists seen for ongoing care and treatment (*n* = 730)		
1–2	447	61.2
3–4	175	24.0
5–6	62	8.5
More than 6	37	5.1
Don’t know	9	1.2
Designated care coordinator (*n* = 742)		
Yes	68	9.2
No	648	87.4
Don’t know	26	3.5
Distance travelled to see medical specialists (*n* = 734)		
<20 km	242	33.0
20–50 km	204	27.8
51–100 km	88	12.0
101–200 km	68	9.2
201–500 km	59	8.0
>500 km	73	9.9
Use telehealth services (*n* = 741)		
Yes	29	3.9
No	697	94.0
Don’t know	15	2.0
Know of a specialist centre for the condition (*n* = 738)		
Yes	163	22.1
No	378	51.2
Unsure	197	26.7
Overall satisfaction with adult health services (*n* = 674)		
Very satisfied	70	10.4
Satisfied	179	26.6
Partly satisfied and partly dissatisfied	255	37.8
Dissatisfied	84	12.5
Very dissatisfied	86	12.7
Had ever used paediatric services (*n* = 746)		
Yes	115	15.4
No	606	81.2
Don’t know	25	3.4
Overall satisfaction with paediatric health services^a^ (*n* = 114)		
Very satisfied	26	22.8
Satisfied	39	34.2
Partly satisfied and partly dissatisfied	37	32.5
Dissatisfied	6	5.3
Very dissatisfied	6	5.3
Satisfied with time between last paediatric and first adult visit^a^ (*n* = 103)		
Yes	51	49.5
No	26	25.2
Don’t know	26	25.2
Problems in transition from paediatric to adult services^a^ (*n* = 108)		
Yes	57	52.8
No	38	35.2
Don’t know	13	12.0

^a^Among respondents who had ever used paediatric services (*n* = 115)

### Experiences of paediatric services and the transition to adult services

Fifteen percent of respondents had ever used paediatric services (Table 8). Just over half of these respondents (57.0 %) were satisfied with the care they had received from paediatric services, while 10.6 % were dissatisfied and 32.5 % were neither satisfied nor dissatisfied. Half of respondents who had used a paediatric health service were satisfied with the time between the last paediatric visit and the first visit to adult services (49.5 %) while 25.2 % were dissatisfied. Half had experienced problems in the transition from paediatric to adult services (52.8 %). Transition is most often defined as “the purposeful, planned movement of adolescents and young adults with chronic physical and medical conditions from child-centred to adult-oriented health care systems” [23].

### Participation in research

Patient registries are sets of clinical and non-clinical data stored in an organised, systemic manner [24]. They can be used to build knowledge about rare disease diagnosis, treatment and management, and enable access to clinical trials of drug treatments and other therapies that are being developed [25, 26]. Only 20.3 % of survey respondents knew of a patient registry for their condition, yet 88.6 % indicated they would join a registry if one existed (Table 9). Of those respondents who know of a patient registry for their condition, 90.6 % had joined that registry.

**Table 9:** Research on condition

	Number of people	% of Response
Would join registry if one existed (*n* = 741)		
Yes	657	88.6
No	13	1.8
Don’t know	71	9.6
Know of a patient registry (*n* = 737)		
Yes	150	20.3
No	587	79.7
Joined a patient registry that they know of ^a^ (*n* = 149)		
Yes	135	90.6
No	4	2.7
Unsure	9	6.0
Would prefer not to say	1	0.7
Informed of clinical trials (*n* = 741)		
Yes	184	24.8
No	515	69.5
Don’t know	42	5.7
Given enough information about clinical trials (*n* = 728)		
Yes	121	16.6
No	487	66.9
Don’t know	120	16.5
Given enough information about research in general (*n* = 735)		
Yes	142	19.3
No	520	70.8
Don’t know	73	9.9
Ever participated in research (*n* = 746)		
Yes	248	33.2
No	479	64.2
Unsure	19	2.5
Type of research participation^b,c^ (*n* = 248)		
Being on a registry	90	36.3
Clinical trial	81	32.7
Recruiting others to participate in clinical trials	17	6.9
Providing samples for research	139	56.0
Patient representative	10	4.0
Survey	25	10.1
Other	27	10.9

^a^Among those who know of a patient registry for their condition (*n* = 150)

^b^Among those who had participated in research (*n* = 260)

^c^Multiple responses reported

One-quarter (24.8 %) of respondents reported they are informed of clinical trials for their condition while fewer agreed that they were given enough information on clinical trials (16.6 %) or on research in general into their condition (19.3 %). One-third (33.2 %) had participated in research into their condition, with the most common form of participation being the provision of biological samples for research, followed by being on a registry and participating in a clinical trial.

## Discussion

Consistent with previous studies conducted in the United Kingdom and Europe [8, 10, 16, 27], around half of the respondents in the current study waited one year or more to be diagnosed with a rare disease, with almost a third waiting five or more years. Two-thirds consulted three or more doctors before receiving a confirmed diagnosis and almost half were given an incorrect diagnosis prior to their final diagnosis. This is problematic in that the absence of an early, accurate diagnosis can contribute to unnecessary or delayed treatment, poorer health outcomes, reduced quality of life, unnecessary hospital admissions and thus inefficient use of health system resources [8, 16, 28–30].

It can be assumed that clinicians work hard to deliver timely, accurate diagnoses for their patients and that a long wait for diagnosis can, at least in part, be attributed to the nature of rare diseases. Most are complex with multi-system dysfunction and thus may require a diagnostic process that involves multiple medical specialties and a systematic method of precluding conditions before arriving at a final diagnosis. However it is also possible that structural features within health systems could contribute to inefficiencies and gaps in the diagnostic process.

Fragmentation and the siloed nature of Australian health systems, organised by medical specialties, could lead to lack of communication between specialties and the experience of patients not being considered as a ‘whole person’, both at the time of diagnosis and during ongoing care and disease management. This possibility suggests that components of the health system need to be better integrated and individual care for people living with rare diseases to be better coordinated [29, 31, 32]. Aligned with this, the role of multi-disciplinary centres of expertise in rare disease requires exploration in the Australian context.

Internationally multi-disciplinary centres have been promoted as mechanisms to bring together health professionals from a range of medical and allied health disciplines. The aim is to provide a team-based approach that integrates services for diseases diagnosis, follow-up and management and enable a continuum of care and care coordination for people living with rare diseases [33–35]. Mental health professionals should be considered a key part of a multi-disciplinary approach, given half of our respondents’ perceived they did not receive sufficient psychological support.

Structural issues may be particularly pertinent for adult health services when compared with paediatric services. The present study found that respondents diagnosed as children were more likely to have been diagnosed within three months, to see fewer doctors before receiving a confirmed diagnosis and they were less likely to receive an incorrect diagnosis. While this could be due to the nature of the diseases and symptoms that onset in childhood compared to adulthood, it could also be due to differences in the structure and operations of paediatric and adult care settings in Australia. Paediatric settings are generally more likely to involve family and carers, have shorter wait times, fewer patients, general paediatricians who cut across specialty areas, multi-disciplinary teams and specialists knowledgeable in rare diseases.

The differences in care settings may also contribute to the problems experienced by a significant number of respondents who needed to transition from paediatric to adult care. Poor transition can influence patterns of integration into adult health services (e.g., not making or keeping appointments) and reduce adherence to treatments, both of which can result in poorer health outcomes [36]. To the extent that there is a lag between the time of transition and when the survey was completed, it is possible that respondent views on the transition experience are associated with recall bias. Thus the reliability of this data must be approached with caution. However, previous Australian findings [37] have also identified the need for improved transition for people living with rare diseases, including better preparation and planning for transition, continuity and coordination of care including the sharing of patient records and medical history, flexibility to allow the involvement of parents and carers and access to transition coordinators.

Health professionals may need greater awareness of rare diseases in order to improve the diagnostic process, and support to meet the information requirements of people newly diagnosed with rare diseases. With 5000 or more rare diseases, it is unrealistic to expect that health professionals would know about every rare disease. However, education could be provided to reinforce to health professionals that when they see a patient whose symptoms they can’t explain, usual practice is to ask whether the cause could be a rare disease. This approach to education seems appropriate since patients have reported that accelerating a correct diagnosis requires the crucial step of recognising their disease is not one frequently encountered but possibly a rare disease [25].

When compared to data on Australians aged 15 years and older from the Australian Health Survey [22], the respondents to the current survey appear more likely to have seen a medical specialist, been an inpatient at hospital, used an outpatient service or used an emergency department. This suggests that health service use may be higher among people living with rare diseases compared to the general population. This claim is supported with a study by Dye et al. (2011) which found that adults living with rare genetic disorders had increased numbers of hospital admissions and longer lengths of stay [38]. Thus the health system costs attributable to rare diseases may be disproportionately high compared to the size of the population living with rare diseases. Information on the impact of rare diseases on the health system is critical for planning services that respond to the needs of people living with rare diseases and epidemiological studies are required to investigate this further.

In this study respondents indicated an overwhelming desire to be involved in research into their condition. In particular, 90 % respondents indicated that they would join a patient registry if one existed for their condition. In Australia, interest in patient registries has grown significantly among clinicians, researchers, industry and patient organisations since registries facilitate research into rare diseases and are particularly important in supporting patient access to local, national and international clinical trials of new drug treatments and therapies [26, 39]. While there are numerous disease specific registries in Australia, these tend to be siloed and lack interoperability. There have been calls for a national registry in Australia for all rare diseases, similar to those developed in other countries [40]. Based on the findings of this study, people living with rare diseases would seem likely to join a disease-specific and/or a national registry, if the existence of such a registry is communicated to them.

It is not possible to determine the generalizability of the results from this study to the total population of people living with a rare disease. There is a paucity of evidence regarding the total number of people living with a rare disease in Australia, and their characteristics such as gender, age distribution, level of education and socio-economic status are unknown. Respondents to this study were self-selected after receiving an email about the survey, most likely from a patient support organisation that was on the distribution list of one or more of the study partners. Thus people without links to a patient support organisation that was known to the study partners will be under-represented in the survey respondents, as will those without a computer/email address and those with lower e-health literacy.

It is assumed that the high proportion of female respondents to the study is over-representative. This may be due to the fact that women are more likely to respond to surveys or the fact that people were recruited to the study through patient organisations to which women may be more likely to belong. While this may bias the survey findings, comparisons to male respondents indicated there were few areas of reported healthcare experiences with significant differences between men and women. An exception to this was the higher percentage of men being diagnosed with a rare disease in childhood and this warrants further investigation.

In relation to age, while it was compulsory for respondents to answer whether or not the person living with a rare disease was aged 18 years or over, it was not compulsory to provide details of actual age in years and relatively few respondents chose to do so (*n* = 257, 31.7 % of the sample). We are not sure why this is the case but as a result it was unreliable to conduct an analysis of survey outcomes by age groups. It is possible that outcomes would be different across age groups, given that health services have likely changed over the course of time, and this could be further investigated.

Sample bias may also exist in relation to the type of diseases which were represented among the study respondents. Some conditions, such as Ehlers-Danlos syndrome, were over-represented in relation to the total sample size. Similarly, it’s possible that more severe disorders, such as those that lead to major incapacity and/or early death, are under-represented. Despite this, survey respondents did represent a range of rare diseases and commonalities in healthcare experiences were identified that cut across disease types. This suggests that considering rare diseases as a collective group is an efficient way for health service providers and policy-makers to respond to the public health issue of rare diseases. In a health system that is oriented towards more common diseases, a collective view should serve to raise the profile of rare diseases and is likely to result in less duplication of efforts and resources across the range of rare diseases.

## Conclusion

This study of 746 survey respondents addresses a gap in the literature regarding the healthcare experiences of Australian adults living with a confirmed rare disease and suggests that not all of their healthcare needs are being met. In line with the study findings, calls for a national plan for rare diseases in Australia [7, 12, 13, 41] have suggested that a national plan should support: patient-centric, integrated, coordinated, multi-disciplinary care; the information needs of people living with rare diseases, their carers, families and health professionals; training for health professionals so that they can better identify rare diseases; and the development of research infrastructure such as patient registries which are easily accessible for people living with rare diseases. The current study provides some much needed evidence to support an emphasis on these issues in national planning for rare diseases in Australia.

### Ethics approval and consent to participate

Ethics approval was obtained from the Western Australian Department of Health, Human Research Ethics Committee (approval number 2014/03).
